# Enhancing Prospective Teachers’ Professional Development Through Shared Collaborative Lesson Planning

**DOI:** 10.3390/bs15060753

**Published:** 2025-05-30

**Authors:** Chen Guo, Xiangdong Chen, Jiawen Chen

**Affiliations:** 1 School of Educational and Psychological Science, Hefei Normal University, Hefei 230601, China; guochentc@126.com; 2 Faculty of Education, East China Normal University, Shanghai 200062, China; 3 School of Artificial Intelligence, Shanghai Normal University Tianhua College, Shanghai 201815, China

**Keywords:** collaborative lesson planning, socially shared regulation of learning, prospective teacher, teacher education

## Abstract

Teacher collaboration, particularly through collaborative lesson planning, plays a key role in fostering professional development and teaching skills. This study investigates the effectiveness of the proposed Shared Collaborative Lesson Planning (SCLP) procedure in enhancing the professional development of prospective teachers through the lens of socially shared regulation of learning (SSRL). Twenty-four prospective information and technology teachers participated in two rounds of SCLP activities. Using a cyclical framework, the prospective teachers engaged in collaborative tasks supported by an online learning platform and other computer-supported collaborative learning (CSCL) tools. Detailed descriptions from observations, recordings, questionnaires, and interviews revealed that the SCLP procedure can help facilitate structured collaboration, improve professional skills, and encourage adaptive regulatory behaviors among the prospective teachers. The integration of CSCL tools further enhanced the effectiveness of the collaborative lesson planning process. However, challenges related to technical proficiency and emotional regulation were identified. The study concludes that the SCLP procedure offers a promising approach for teacher education by fostering a collaborative learning environment and promoting regulatory behaviors. Future research should explore the long-term impacts and adaptability across diverse educational contexts to strengthen teacher education programs or courses.

## 1. Introduction

Teacher collaboration is essential in fostering professional development and enhancing teaching effectiveness. Collaborative lesson planning has evolved as an important approach for promoting teacher professional skills and teaching performances in teacher education programs, particularly for the prospective teachers (Bauml, 2014; Gutierez, 2021; Ruys et al., 2012). This approach not only fosters a sense of community among future educators, but also plays a crucial role in enhancing their professional development (Backfisch et al., 2023). By engaging in group work to analyze instructional tasks, design lesson plans, conduct teaching sessions, and reflect on their teaching practices, prospective teachers can be prepared to plunge into classroom teaching more effectively (Voogt et al., 2015).

One of the key purposes of teacher collaboration is to establish communication, collaboration, and coordination among teachers (Vangrieken et al., 2015). This can be achieved by the lesson planning groups, where teachers work together based on shared instructional goals to accomplish shared lesson planning outcomes. First, collaborative lesson planning is inherently a social process. Normally, a lesson planning group is composed of teachers with common lesson planning tasks, and they can jointly discuss and interact with each other to improve lesson planning outcomes (Ruys et al., 2012). Second, collaborative lesson planning involves the process of regulatory behaviors and performances. Teachers can not only regularly collaborate and exchange ideas and insights, which can improve their teaching in this process, but are also required to integrate, evaluate, and collaborate with others in the lesson planning groups (Gutierez, 2021). Furthermore, the collaborative lesson planning process requires necessary coordination in attitudes and awareness from teachers who have a strong sense of synergy and collaborative skills (Yuan & Zhang, 2016).

A theory that is highly applicable to collaborative lesson planning is social shared regulation of learning (SSRL). It examines how members in a planning group collectively regulate the learning process (Hadwin et al., 2017). SSRL occurs when individuals work together as a whole to regulate their learning, such as when the group works together to formulate the same learning tasks and goals, plan jointly, monitor each other, and construct shared evaluation mechanisms (Järvelä et al., 2013). SSRL is in fact a key competency in the collaborative learning process, as collaboration implies that the groups work together to construct shared task goals, cognition, and strategies, while regulating learning through the monitoring and control of meta-cognitive aspects, such as cognition, affect, and motivation (Panadero & Järvelä, 2015).

### 1.1. Collaborative Lesson Planning

Collaborative lesson planning can be framed as the process in which teachers of the same subject content area or grade level prepare lessons for a number of curriculum areas and subsequently implement the planned lessons in the classrooms (Alloway, 2014). When teachers collaborate on lesson preparation, they can work together to design and develop shared lesson plans for specific teaching goals and tasks, and attempt to improve the delivery of lessons through a cyclical process of instructional design, trial teaching, observation, and mutual evaluation (Ruys et al., 2012). Collaborative lesson planning affords prospective teachers an avenue to share knowledge and experiences that might result in significant enhancements of their competencies in lesson design (Bauml, 2014). Collaboration among peers enables the exchange of diverse ideas and strategies, allowing individuals to learn from each other’s successes and challenges. The shared learning experience is invaluable in building a robust foundation for future professional development (Pham et al., 2023). Another advantage of collaborative lesson planning is that it is well-structured. The prospective teachers can approach the shared goal setting and collaborative reflection in a structured manner, whereby aspects of their lesson plans and teaching methods are systematically dealt with (Graham, 2007). Such a structured process contributes positively to the learning of the prospective teachers, as it promotes critical thinking and self-assessment (Gutierez, 2021). Through this collaboration process, they not only obtain suggestions of their performances from their group members, but also an overview of a variety of teaching methods that allows them to develop professionally (Thobela et al., 2023). Additionally, the integration of technology into the collaborative lesson planning process has further enhanced the learning experience. König et al. (2024) and Widjaja et al. (2021) provided evidence of how computer-supported collaborative learning (CSCL) tools allowed prospective teachers to collaborate with each other on lesson planning, give feedback in real time, and provide a variety of resources that will add depth to their instructional design.

However, while the positive aspects of collaborative lesson planning seem evident, it is necessary to recognize the challenges that go along with it. One of the major challenges involves balancing the lesson planning processes among the group members. Achieving a fair division of labor may be difficult, as individual workloads may vary (Fletcher & Beckey, 2023). This imbalance can lead to frustration and resentment among the members, ultimately undermining the spirit of collaboration that collaborative lesson planning is meant to foster. In addition, challenges such as time management, professional responsibility, and a lack of a collaborative atmosphere can hinder the successful design and implementation of such a collaborative approach in teacher education (Rahman, 2019; Vangrieken et al., 2015).

The processes of collaborative lesson planning have been described differently across various studies (Alloway, 2014; Bauml, 2014; Gan & Qian, 2016; Gutierez, 2021; Li & Zhao, 2011). Li and Zhao (2011), for instance, described an overview of a common model of lesson planning that puts emphasis on personal lesson planning coupled with collective discussion and reflection. The model demonstrated how personal reflection and discourses of collaboration balance in honing the practice of teaching. Building on this, Gan and Qian (2016) identified specific stages of collaborative lesson planning that include individual planning, discussions among peers, and group evaluation of the planned lessons. Their emphasis on the evaluation underlined an important step in the process, since prospective teachers can assess the practicality and effectiveness of their lesson design collaboratively. This iterative approach can foster professional growth, and encourages teachers to consider diverse perspectives.

In other related studies, Alloway (2014) explored the significance of teacher collaboration and outlined stages that consider cultural and curriculum norms, as well as mediation strategies. The author stated that collaborative lesson planning is not just about the mechanics of lesson design, but about navigating the broader context in which teaching occurs. Understanding cultural nuances and curriculum expectations is key to collaboration for prospective teachers, Davidson et al. (2023) described and discussed an approach to collaborative mathematics lesson planning that helped prospective teachers use their planning time in a productive and effective way. They found that subject-specific considerations can further enhance the effectiveness of collaborative planning, enabling educators to tailor their discussions and strategies to the unique demands of different content areas.

### 1.2. Socially Shared Regulation of Learning (SSRL)

When individuals work collaboratively, at least three types of regulated learning come into play (Järvelä & Hadwin, 2013): (1) each group member takes responsibility for regulating his or her learning (self-regulated learning), (2) each group member supports peers in regulating their learning (co-regulated learning), and (3) the group comes together to collectively regulate learning processes in a synchronized manner (shared regulation of learning). What is important and different in the shared regulation of learning is that self-regulated learning theory extends conceptions of learning beyond cognitive processes and outcomes, acknowledging the interactive roles of motivation, emotion, meta-cognition, and strategic behavior in successful learning (Zimmerman & Schunk, 2011). SSRL refers to processes by which group members regulate their collective activity. This type of regulation involves interdependent or collectively shared regulatory processes, beliefs, and knowledge (e.g., strategies, monitoring, evaluation, goal setting, motivation, and meta-cognitive decision making) orchestrated in the service of a co-constructed or shared outcome (Hadwin et al., 2017). According to Järvelä et al. (2016), SSRL consists of four core stages, including understanding, planning, monitoring, and evaluation. These stages facilitate interaction and mutual understanding among group members, enabling learners to manage their learning processes and coordinate and reflect within the team through shared goals and tasks.

Previous research identified a positive relationship between socially shared regulatory behaviors and the quality of collaborative learning (e.g., Hurme et al., 2009; Järvelä et al., 2023; Lee et al., 2015; Malmberg et al., 2015; J. Zheng et al., 2019). For instance, Hurme et al. (2009) investigated the process and effectiveness of regulation of learning in group task understanding and in a problem-solving setting. They found that SSRL happens when a group member regulates a group’s problem-solving process and the other group members react to the initiative. Malmberg et al. (2015) explored how groups progress in their SSRL in a CSCL context, and found that groups with better collaborative learning performances mainly focused on regulating the cognitive, motivational, and social aspects of collaboration. Similarly, J. Zheng et al. (2019) conducted a chain analysis on the online chats and logs of students completing a learning task in a CSCL environment. They found that the successful groups were most likely to start with self-executing and end with socially shared monitoring. Furthermore, Nakata et al. (2022) explored how certain class modes of regulation emerge in the context of collaborative learning. They found that socially shared regulation among students is critical for creating a positive classroom environment in which students influence and motivate each other.

Meanwhile, SSRL and collaborative practices in teacher education have been widely examined, and their crucial role in enhancing teachers’ professional development is underlined. Isohätälä et al. (2017) investigated how SSRL among six groups of prospective teachers emerged during the fluctuation of participation in interaction in collaborative learning. The results showed that SSRL involved more active participation than task-focused interaction overall, and that it often coincided with increases in participation to a higher level. Voogt et al. (2015) investigated collaborative lesson planning engagement with a group of teachers, and identified issues such as time constraints and inequity of participation, which suggest structured facilitation in the activities of collaborative lesson planning. In recent studies, Sulla et al. (2023) indicated that SSRL can promote knowledge analysis and task reflection through team collaboration, enhancing individual understanding of the learning process and collaborative learning abilities. Y. Zheng et al. (2024) examined the impact of SSRL strategies in a quasi-experiment with 48 student teachers over a semester and found that SSRL significantly improved collaborative reflection ability, especially in critical reflection, and provided insights into its influence across different learning stages.

Though the importance of SSRL has been recognized, there is still limited understanding about how prospective teachers practically engage in social forms of regulatory processes and to what extent the regulatory processes can influence their learning (Järvelä et al., 2016; Ortube et al., 2024; Ucan & Webb, 2015; Y. Zheng et al., 2024). Meanwhile, only a few studies attempted to investigate how prospective teachers implement SSRL to promote collaborative learning and achieve shared goals with group members (Näykki et al., 2017; Zhang et al., 2021). As the collaborative lesson-planning activities designed in this study require a high quality of collaboration and interaction among the prospective teachers, and the activities can primarily be conducted and controlled by the groups, regulation of learning is indispensable. One of the prominent features of the collaborative lesson planning procedure proposed is that prospective teachers should be guided to regulate and monitor each other to ensure the processes of the tasks. Meanwhile, as they were in the same subject teaching group and have shared goals and requirements, they can work more efficiently when socially shared regulatory behaviors occur in their collaborative learning and knowledge construction process.

### 1.3. The Shared Collaborative Lesson Planning (SCLP) Procedure

The regulation of learning is characterized by four key features. It is intentional and goal-directed (Järvelä & Hadwin, 2013), as it is influenced by various factors such as content, level, and difficulty (Zimmerman, 2008). It encompasses meta-cognitive elements such as planning, monitoring, and controlling processes, which can occur at individual or group levels (Hurme et al., 2006). Additionally, it involves cognitive, behavioral, and emotional regulation, focusing on monitoring and controlling thinking, beliefs, and strategies to achieve goals, rather than merely constructing knowledge (Järvelä & Hadwin, 2013; Järvelä et al., 2023). The social aspect is also important, as learner interactions are vital for regulation of learning (L. Zheng et al., 2020), with research emphasizing the impact of environmental and social factors on learning (Hadwin et al., 2017). These features are synthesized with collaborative lesson planning, whose process is inherently goal-directed, as teachers in a group share common instructional goals and plan lessons based on the same teaching objectives, curriculum standards, and resources, ensuring alignment towards a shared goal. It also involves meta-cognitive, cognitive, and affective behaviors, requiring teachers to select appropriate strategies to regulate cognitive, affective, and motivational aspects to adapt to the current state of learning and move towards the completion of shared goals. Furthermore, the social nature of regulation manifests in the dynamic exchange of diverse pedagogical perspectives during collaborative lesson planning, fostering collective growth through negotiated decision-making and iterative refinement of instructional designs.

Collaborative lesson planning offers an opportunity for communities to generate professional knowledge through interaction, which fosters the creation of new understandings (Gutierez, 2021). According to Wenger (1998), learning occurs everywhere, and is a social process of participation. Learning also involves becoming part of a “sustained community of practice”—a group of individuals engaged in a “shared domain of human endeavor” (Farnsworth et al., 2016). In collaborative lesson planning, communities of practice encompass “groups of people who share a concern, a set of problems, or a passion about a topic, and who deepen their knowledge and expertise in this area by interacting on an ongoing basis” (Wenger et al., 2002).

Collaborative lesson planning involves different levels of regulation. First, it requires cognitive level regulation. Previous research indicated that promoting teachers’ professional development through collaborative lesson planning hinges on providing platforms or tools for exchanging teaching experiences and receiving feedback from colleagues (Bauml, 2014). Given that prospective teachers often have diverse professional backgrounds, subject knowledge, teaching experiences, and teaching styles, they may hold different understandings of instructional goals, teaching resources, instructional design, and lesson planning tasks. Therefore, each should take responsibility for monitoring, evaluating, and adapting the contributions of other group members to ensure lesson planning progress. Second, collaborative lesson planning involves meta-cognitive level regulation. Unlike individual lesson-planning activities, this process requires structured reflection on how the lesson plan is developed. The reflection may take the form of simulated teaching, lesson plan presentations, peer observation, and evaluation. Through the processes, teachers’ meta-cognitive thinking can be improved, and they can largely contribute to the reflection and adaptation of shared lesson plans. Third, maintaining a high level of participation and collaboration in the lesson planning process requires necessary regulation of motivation and emotion. Previous research emphasized that trust and confidentiality form the foundation for effective learning and growth of prospective teachers within a teacher development community (Liou et al., 2017). Furthermore, effective collaboration during lesson planning can lead teachers to develop a positive sense of group efficacy, which can alleviate the nervousness and uncertainty arising from the lack of teaching experience in prospective teachers (Goddard et al., 2015).

The core of SSRL activities includes a range of meta-cognitive processes, such as task understanding, goal setting, planning, monitoring, evaluation, and adapting (Hadwin et al., 2017; Malmberg et al., 2015). Previous studies (Alloway, 2014; Cajkler et al., 2014; Smit et al., 2018) indicated that the typical procedure for collaborative lesson planning can be summarized in three main stages: Lesson topic identification, joint coordination, and simulated teaching and evaluation. In the first stage, the prospective teachers in a group should be responsible for the identification of topics for upcoming lessons, establish shared curriculum standards, and determine key learning points. They normally obtain a thorough understanding of the lesson topic to develop a common lesson plan and a schedule. The second stage, joint coordination, involves meta-cognitive, cognitive, and affective coordination, especially when differences in the ideas and understanding emerge during the collaboration. The prospective teachers can use regulatory strategies to modify individual and collective engagement and learning. Additionally, they can obtain information about the collaboration and interaction of group members as well, such as the individual and collective progress of task completion. In the third stage, the teachers normally need to conduct structured or repetitive simulated teaching practices, a lesson plan demonstration and illustration, peer observation, and mutual evaluation. A main advantage of collaborative lesson planning is that the final lesson plan can be refined through the systematic evaluation and advice of modification proposed by others in the community.

**Figure 1:**
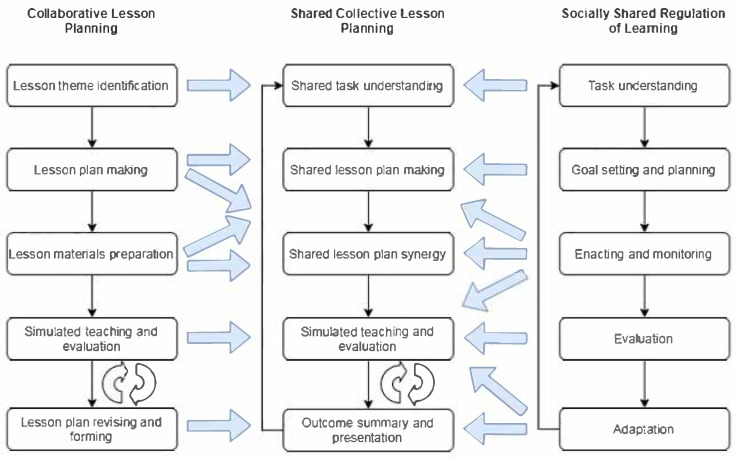
The relationships between collaborative lesson planning, the SCLP procedure, and SSRL.

Therefore, drawing upon the theoretical foundations outlined earlier, the SCLP procedure underpinning this study is proposed. It integrates the levels of regulation strategies—cognitive, meta-cognitive, motivational, and emotional—with the key shared regulatory processes in learning, including task understanding, planning, monitoring, evaluation, and adaptation, within the context of collaborative lesson planning. The relationships among the components in collaborative lesson planning, socially shared regulation of learning, and the SCLP procedure is depicted in Figure 1. It consists of five key stages: shared task understanding, shared lesson plan creation, shared lesson plan synergy, simulated teaching and evaluation, and outcome summary and presentation. The structure of the procedure is cyclical, and teachers involved in the SCLP can return to any of the previous stages to optimize teaching and planning during collaborative lesson planning activities. For instance, the fourth and fifth stages may include a series of repetitive activities with a variety of regulatory behaviors and strategies.

### 1.4. Research Goal and Questions

This study employs a qualitative approach, utilizing observations, recordings, questionnaires, and interviews to gather data on the SCLP activities. The SCLP procedure involves a cyclical framework that can enable prospective teachers to engage in structured collaborative tasks. The primary goal of this research is to investigate the effectiveness of the SCLP procedure in enhancing prospective teachers’ professional development, with a specific focus on how SSRL and CSCL tools facilitate structured collaboration, adaptive regulatory behaviors, and teaching skill improvement. Two main research questions are included: (1) How did involvement in the SCLP procedure impact prospective teachers’ collaborative learning, professional development, and adaptive regulatory behaviors? (2) How did shared regulation and CSCL tools help support prospective teachers’ collaborative lesson planning and teaching practices?

## 2. Materials and Methods

### 2.1. Participants

Participants of this study were 24 graduate students majoring in modern instructional technology from a normal university in China. These students would be middle school information and communication technology teachers when completing their graduate program. They took part in a required twelve-week teacher education course. During the first six weeks, the instructor delivered a series of lectures, providing educational materials and instruction on topics related to the development of prospective teachers’ teaching skills and content knowledge. In the subsequent six weeks, they engaged in two rounds of collaborative lesson planning activities to train their teaching skills and competencies in the micro-teaching classrooms. Prior to the SCLP activities, participants were self-organized into five groups, each consisting of four or five members. The course instructor presented video cases of collaborative lesson planning activities from other teacher training programs available online. The prospective teachers were then encouraged to engage in a series of lesson planning activities, with a focus on mutually regulating the teaching and learning to ensure the successful completion of the tasks. Their performances and behaviors in the micro-teaching classrooms were recorded, and served as the primary data source. Informed consent was received, and all agreed to be enrolled and recorded in the study. At the end of each round of lesson planning activities, each group constructed a group shared lesson plan as the main outcome. Although the teachers were primarily responsible for completing the lesson planning tasks independently, the instructor and the investigator provided timely support and feedback whenever they needed help, or were faced with difficulties in making progress with their work.

### 2.2. Online Collaborative Learning Platform for Supporting the SCLP

**Figure 2:**
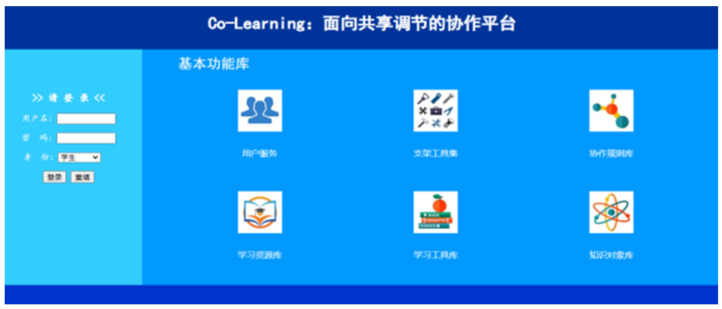
The front page of the collaborative learning platform.

An online collaborative learning tool, *Co-Learning Platform*, was utilized as the primary CSCL tool for supporting collaborative lesson planning. This platform was developed by our research team. The platform offers four types of user access: Student, teacher, administrator, and visitor. As the participants were graduate students, they received training on how to use the platform and were assigned a student account one week prior to the commencement of the SCLP. The platform includes three key features from the student side. First, the embedded collaboration scripts functioned as the scaffolds, providing guidance to participants as they completed lesson planning tasks during collaboration and regulation (Vogel et al., 2022). For example, in the shared task understanding stage, a *Group Task Understanding Questionnaire* (Appendix A) was provided to each participant as the micro-script (a type of scaffold that guides learners through sequential steps of productive collaboration via prompting learners to follow predetermined collaborative actions) to enhance their understanding of the tasks and activities. In the shared lesson plan synergy stage, participants can bridge the gap between individual and group task understanding by reviewing the questionnaires submitted by both themselves and the group members. Second, the platform includes group awareness tools that allow participants to access individual and collective information relevant to the lesson planning progress. For instance, after a group completes the *Individual Initial Status Questionnaires* (Appendix B) during the shared lesson plan making stage, a radar, bar or pie chart is generated, displaying each participant’s interests, perceptions of challenges, and level of involvement in specific activities. This information is visible to all group members. Third, the platform facilitates communication and interaction between individual students, group members, and the course instructor, which can be crucial for effective shared regulation of learning. Figure 2 displays the front page of the *Co-Learning Platform*. After logging in, the participants can select functions from the left-hand menu, which include access to the course and group member information, instructor feedback or prompts, and micro-scripts that guide the collaborative lesson planning and simulated teaching activities.

### 2.3. Procedure

Before engaging in the collaborative lesson planning activities, participants divided themselves into five groups, and each group gathered around a table to establish an initial rapport and connection. Subsequently, they formed a chatting group with a widely-used instant messaging application, WeChat. The functions of the chat group included organizing and managing learning activities, facilitating communication and sharing, distributing and submitting files, and offering information and feedback. They were then involved in two rounds of SCLP activities, which followed a procedure consisting of five stages (Figure 1): (1) Shared task understanding, (2) shared lesson plan development, (3) shared lesson plan synergy, (4) simulated teaching and evaluation, and (5) outcome summary and presentation. At each stage, both the instructor and the researcher provided targeted tools or interventions.

#### 2.3.1. Shared Task Understanding

Task understanding involves recognizing and comprehending tasks through an analysis of the characteristics, considering cognitive, meta-cognitive, and emotional aspects, as well as activating prior knowledge (Zimmerman, 2008; Winne & Hadwin, 1998). Shared task understanding requires group members to collaborate in achieving a consistent understanding of the assigned tasks or given situations. This represents the initial and most critical stage in the shared regulation of learning, laying a foundation for goal setting, planning, and subsequent regulatory processes.

At the initial stage of the SCLP process, the course instructor assigned lesson planning tasks and requirements to the prospective teachers. Each group was asked to design and develop a shared lesson plan based on the units and lessons from the middle school information technology textbooks. Each participant needed to engage in the simulated teaching practices within the group, focusing on the group lesson plan. To facilitate the development of a shared understanding of the lesson planning tasks, the participants were encouraged to work together to determine the lesson topics, negotiate the task characteristics (e.g., learning activities, task completion time), cognitive content (learning objectives, content, criteria), and other key aspects (task goals, learning strategies, available instructional resources). A group understanding micro-script tool that can support the groups in reaching a consensus regarding the lesson planning tasks was provided to the participants. The tool not only helped them understand the lesson content and topics, but also provided a channel for understanding each other’s teaching knowledge and skills. By the end of this stage, each group had developed a shared understanding of the task characteristics, the lesson topic, and the teaching and learning context for future practice.

#### 2.3.2. Shared Lesson Plan Making

**Figure 3:**
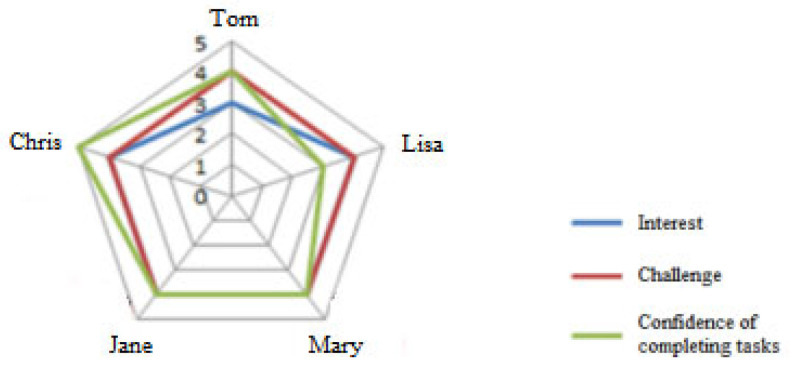
Radar graph of the emotional and motivational state of one group.

At the beginning of this stage, participants were asked to report their individual emotions, interests, awareness of challenges, and self-efficacy regarding the tasks and activities on the *Co-Learning Platform*. Responses were visually represented through radar, pillar, or pie charts accessible to the groups (Figure 3). This information served as a valuable reference for facilitating discussions on key aspects of the SCLP, including role assignment, duty and resource distribution, lesson plan editing and modification, and shared evaluation criteria formulation. During the collaborative lesson planning process, participants gained a deeper understanding of the tasks through the micro-scripts and group awareness tools available on the online platform. These micro-scripts help guide the participants in breaking down a general task into several sub-tasks, such as defining specific learning goals, selecting teaching and learning resources, and determining the appropriate time and context for pedagogically integrating CSCL tools. Furthermore, each group engaged in a detailed division of labor based on the tasks. At the end of this stage, each group developed a time schedule for the co-construction of the shared lesson plan.

#### 2.3.3. Shared Lesson Plan Synergy

The shared lesson plan synergy stage is an essential component of the SCLP procedure. From the perspective of SSRL, each teacher is expected to select appropriate regulatory strategies to enhance and manage both their learning and their collaboration with group members. These strategies encompass a series of interaction and coordination efforts which aim to develop a group lesson plan. Each group was guided to use *Shimo*, a cloud-based online collaborative documentation tool, to create the shared lesson plan. This tool functions similarly to platforms like *Google Docs* and *Quip*, enabling group members to view and edit the lesson plan documents synchronously. By using this tool, members can observe the actions of other members, track modifications, and review operational records. Furthermore, each prospective teacher was assigned the responsibility of monitoring the participation and learning status of others, tracking the group progress of collaborative learning, and evaluating the outcomes of the group lesson plan. This monitoring process helps identify potential issues and discrepancies between the current progress and the intended goals. To facilitate these tasks and ensure effective process monitoring, a *Group Interaction Monitoring Template* was developed and distributed to the participants. This template includes questions across the four dimensions—cognitive, meta-cognitive, monitoring, and motivational—that prompt each participant to notice the interactions and contributions of the group members.

#### 2.3.4. Simulated Teaching and Evaluation

Once finishing the initial version of shared lesson plan, the groups organized a schedule for each member to carry out in-group simulated teaching sessions and engage in mutual evaluation. The teaching practices were conducted in the micro-teaching classrooms equipped with various instructional tools, such as personal computers, projectors, interactive whiteboards, and video or audio equipment. Each teacher delivered the shared lesson plan at the podium. The other group members observed the simulated teaching and provided feedback and suggestions. The teaching can help improve participants’ teaching and presentation skills. Additionally, each participant was required to review and evaluate the organization and content of the shared lesson plans. Modifications to the shared lesson plan were made, which also facilitated reflections on the SSRL process and teaching performance in the SCLP. This allows the prospective teachers to remind their peers to make necessary changes when task understandings, lesson plans, teaching goals, and regulatory strategies were deemed ineffective for the current or future tasks.

#### 2.3.5. Outcome Summary and Presentation

The final shared lesson plan was the primary product of the SCLP activities for each group. Other types of the outcomes include content-related materials, newly-developed websites or applications, and instructional artifacts. Participants were encouraged to continuously revise and refine the shared lesson plan and other related outcomes before submitting the final version. Throughout this process, a series of simulated teaching practices, group evaluation, and discussions can enable each group to select a representative to present the group shared lesson plans to the entire class. In the context of collaborative lesson planning, group reflection facilitates the sharing of diverse perspectives, enabling students to explore the pedagogical and curricular dimensions of their planning. By engaging in structured reflective activities, prospective teachers can critically evaluate the quality of their lesson plans, discuss alternative solutions, and reconsider whether and how to integrate CSCL tools in teaching. Before the final presentation, the group representative and other members engaged in a “practice-evaluation-adaptation-practice” training cycle to improve the quality of the shared lesson plan and their teaching performances. After the presentations, each prospective teacher conducted a group reflection regarding the individual and other group members’ contributions, motivations, and challenges across the overall SCLP procedure and the regulatory processes.

### 2.4. Data Collection and Analysis

This case study mainly collects and analyzes data from class observations, audio or video recordings, interviews, and the micro-scripts on the *Co-Learning Platform*. To investigate how the prospective teachers interact and collaborate with others for lesson planning and their SSRL behaviors and performances during the SCLP, qualitative data were recorded using the classroom monitors or other mobile devices provided by the investigator and the course instructor. These recordings were subsequently uploaded to a cloud storage system for safekeeping and later analysis by the investigator. Transcriptions of selected segments of the discourse from participant interactions were edited and saved into Word documents. Relevant quotations or dialogues are presented to illustrate participants’ collaboration and regulation with others.

### 2.5. Ethical Considerations

This study received approval from the Institutional Review Board (IRB) of the normal university involved. Before the data collection process, the investigator provided participants with a detailed explanation of the study’s purpose, the data collection process, and the potential risks and benefits. Participants’ rights were fully protected in accordance with IRB guidelines, and written consent was obtained from all participants. Participants were informed that they had the right to withdraw from the study at any time without any repercussions or loss of benefits. Throughout the data collection and analysis stages, all participant information was anonymized and securely protected.

## 3. Results

### 3.1. The First Round of SCLP

#### 3.1.1. Shared Task Understanding in the First Round

At the beginning of the course, the course instructor assigned the first instructional design task for the prospective teachers: each group was required to work together to determine a lesson topic, then design and develop a 45-min lesson based on the curriculum standards and current information technology subject textbooks. When completing the course design, each participant needed to conduct a simulated teaching practice within the group based on the group’s lesson plan.

Once the task was announced, each group began to analyze the task and plan for the lesson in the micro-teaching classroom. The course instructor and teaching assistants provided guidance and inspection as needed. Each group determined the lesson topic within two days. For example, one prospective teacher noted in the *Group Task Understanding Questionnaire*:

Through rounds of group discussion and negotiation, we chose machine learning as our lesson topic because three group members had studied it in high school.

The principle for lesson topic selection of this group was based on familiarity with the content. This approach can minimize the time needed for learning new materials and keep them within their comfort zone. Another group noted:

We chose the lesson topic *Experiencing Computer Vision* for three reasons. First, we share a common interest in the topic. Second, artificial intelligence is currently a hot topic in our lives. Third, we treat it as a challenge that would help us improve our information technology knowledge and teaching skills.

Rather than drawing from prior learning experiences, this group determined their lesson topic primarily based on their shared interests as well as contemporary hot-debated topics. The members of this group demonstrated an intrinsic desire to meet challenges and to learn new things.

One major part of the task understanding stage was breaking down the “big” lesson planning task into “small” subtasks. One way to divide the work was based on the production of the outcomes or artifacts. For example, Participant A was assigned to create a PowerPoint presentation, and Participants B and C were responsible for editing and refining the shared lesson plan, while the other members worked on designing teaching evaluation standards. Another way was to divide the task based on the main components of a complete lesson plan. The second group decomposed their work into sections such as teaching content and goals, learning materials and settings analysis, teaching methods and activities design, instructional resources design and application, and the scaffolding of instruments and tools. The group then negotiated on how to distribute these subtasks, with each member choosing a role and the corresponding work. This was slightly different from the second round of this stage, where the course instructor and the investigator suggested the participants to consider each member’s strengths and preferences when allocating roles and tasks.

By the end of this stage, each group generally reached a consensus on the key elements of the task, such as the topic for the lesson plan, expected teaching resources, supporting instructional technologies, and the logic and structure for teaching. They developed standardized and detailed course goals and content, laying a solid foundation for the following SCLP procedure.

#### 3.1.2. Shared Plan Making in the First Round

At this stage, participants engaged in continuous collaboration to develop the shared lesson plan. They were instructed to complete an *Individual Initial Status Questionnaire* (Appendix B) on the online collaborative learning platform, where they provided insights into their individual interests, awareness of challenges, and self-efficacy regarding the activities and tasks. Figure 3 shows the initial levels reported by a group in each of the dimensions (members’ names are anonymized).

Meanwhile, each prospective teacher (PT) expressed their preferences for the desired group dynamic for executing the tasks within the SCLP procedure. As members in one group noted:

- PT A: I wish a comfortable and efficient atmosphere where everyone can contribute effectively to the SCLP.
- PT B: Each member should be able to share their ideas and perspectives without any concerns. The group leader should facilitate the progress of collaborative learning and lesson planning activities, while another member should be responsible for documenting important discussions and events.
- PT C: I hope each member can freely express their opinions and listen to others with an open mind, contributing to a healthy collaborative learning environment.
- PT D: Everyone should have the opportunity to speak out their own views on the topics and issues.
- PT E: I wish a harmonious, relaxed, and efficient collaborative learning and teaching atmosphere.

When making the initial lesson plan, group members worked intensively with each other. Key topics such as the instructional goals, evaluation criteria for the shared lesson plan, and the detailed arrangement of time and tasks were given considerable attention and raised in-depth discussions. For instance, the *Experiencing the Computer Vision* group arranged two morning sessions to confirm the main goals of the lesson planning tasks. The agreed goals included:

1. Co-construct a complete shared lesson plan.
2. Prepare a lesson plan presentation document.
3. Collect and process accessible teaching resources.
4. Improve collaborative lesson planning and teaching skills.
5. Achieve the shared goals, with each member presenting the lesson plan in a personalized manner.

Additionally, the group carefully reconsidered the task assignments and the responsibilities of each member. They then developed a concrete plan and a time schedule conducive to the implementation of the main task for the SCLP. The other groups followed a similar procedure for lesson plan making. However, the reports of the affective and motivational status reflected that there was still a large difference in the interest and efficacy among the members, and the groups did not pay due attention to this difference during the lesson planning process, which also indicated the subsequent need to strengthen the prospective teachers’ support for the perception and regulation of the group’s affective and motivational status.

#### 3.1.3. Shared Lesson Plan Synergy in the First Round

At this stage, the prospective teachers began to engage in deep discussions and collaboratively develop lesson plans through face-to-face or online interactions while also preparing relevant teaching materials. They actively performed a variety of regulatory behaviors and strategies. A significant advantage of using the regulatory strategies was that they helped the participants focus on the tasks and ensured they kept up with other members. A classroom observation field note by the investigator recorded:

- When one group member was distracted by the rain outside the window, another member gently reminded them to refocus on the group discussion and summarized what had been discussed.

In another similar case, a participant was trying to monitor and regulate another absent-minded member (transcription from a piece of recording):

- PT A: We can go through the whole process and divide it into parts. Then we each choose a part to write.
- PT B: Do we need to create a file on the online collaborative documentation platform (*Shimo*)?
- PT A: Of course.
- PT C: was watching a video on her mobile phone.
- PT D: Please stop watching.
- PT E: There is one more document to write, but we cannot finish it now.
- PT B: Let’s concentrate on working out the lesson plan first.
- PT C: Sure, let’s get started.

For each teacher, the mutual interaction and perceived contribution with others varied. Therefore, the differences in communication and interaction among members could result in uneven participation and inefficient collaborative learning in the SCLP tasks and activities. Through the scaffolding of the micro-scripts (*Group Interaction Monitoring Templates* and other questionnaires) via the *Co-Learning Platform*, participants can perceive the role each member played in facilitating learning and interaction. They also began to reflect on how to better collaborate with and regulate each other. Table 1 shows a sample of the interaction monitoring template submitted by one participant. In fact, the synergy and shared regulation between participants were evident at each stage of the SCLP.

#### 3.1.4. Simulated Teaching and Evaluation in the First Round

**Figure 4:**
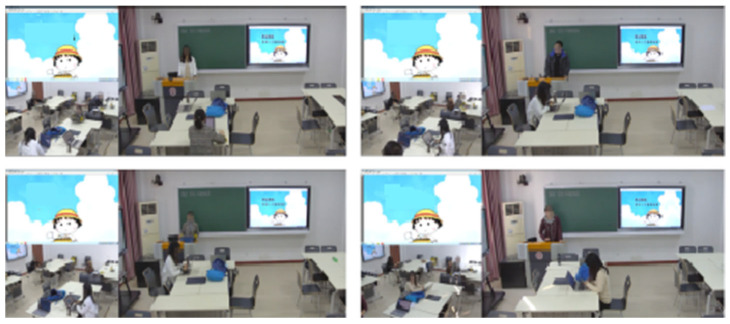
The simulated teaching practice within a group.

Through discussions and collaborative learning, each group developed an initial draft of a shared lesson plan. Subsequently, they arranged time for the simulated teaching practice within the group. While with the same lesson plan, each member can present and teach based on the material in a personalized manner. Figure 4 shows that each participant presented the shared lesson plan to the other group members.

When each participant finished a simulated teaching, the other members orally evaluated the simulated teaching performance of each member of the group, and they commented on the strengths and weaknesses of the teacher’s performance, as well as the problems of the lesson plan. Informed by the evaluations and suggestions, the participant was able to engage in self-reflection, critically reviewing, summarizing, and adapting the teaching performance in preparation for future practices. Meanwhile, the group worked collaboratively to refine the shared lesson plan, incorporating insights from the suggestions and outcomes. Finally, all the groups co-constructed the final version of the shared lesson plans.

#### 3.1.5. Outcome Summary and Presentation in the First Round

**Figure 5:**
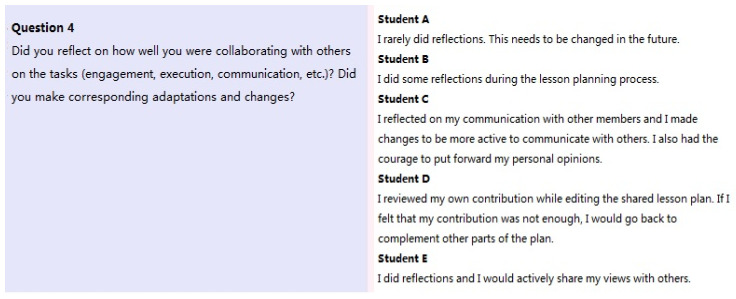
Group responses of a question in the *Reflection on the SCLP Procedure*.

In the final stage of SCLP, each group developed a completed shared lesson plan and relevant teaching materials. One group even designed a graphic poster to visually represent the complex logic structure of the lesson plan. A representative from each group utilized the interactive whiteboard to present the main content of the group lesson plan to the whole class. After the presentations, a *reflection on the SCLP procedure* (Appendix C) was provided and sent to the participants. This micro-script was designed to help the participants reflect on the performances and the challenges they faced in this round of the SCLP. Participants could view the responses from other members on the *Co-Learning Platform*. Figure 5 displays a group member’s responses to a question from the micro-script (the text has been translated into English).

#### 3.1.6. Summaries on the First Round of SCLP

After the outcome summary and presentation stage, participants were asked to reflect on their collaborative learning and regulatory behaviors during the previous SCLP activities. They identified the major challenges that the group encountered, and discussed the strategies employed to address them:

- Group A: Due to unfamiliarity with the lesson content, we encountered difficulties in integrating the principles of teaching with content knowledge, such as how to enable teachers to adhere strictly to the instructional norms and at the same time have a high level of mastery of subject knowledge. To address this problem, our group tried to divide the tasks to allow each member to focus on specific areas of content. However, there were arguments regarding the logical order in which the knowledge points should be organized, which led to delays in lesson planning. Some group members also strayed from the topics. Finally, we reached a consensus on the lesson topic after extensive discussion and negotiation in the second stage of SCLP.
- Group B: Our group encountered several technical challenges due to a lack of sufficient supporting materials. We had to search for the packages and related materials by ourselves. Two members took primary responsibility for gathering the items and managed to obtain the required information. Subsequently, our group had a discussion to determine the best way to implement the project.
- Group C: Determining the lesson topic proved to be a challenge, as five members suggested five lesson topics. We initiated several discussion activities to finally confirm. Another issue was related to the hardware failure. The recording and playback equipment in the micro-teaching classroom unexpectedly malfunctioned, and it took much time to coordinate with the technicians to resolve the issue.

SSRL offers valuable insights into how groups perceive problems during reflection and the role collaboration plays in these situations. While each group identified challenges, the most effective approach for overcoming the difficulties involved collaboration and open discussion. This reflects a core characteristic of the SCLP, which emphasizes the central role of collaborative learning and negotiation among members.

Another important point in group reflections is about how prospective teachers recognize and address disagreements that arise during lesson planning and collaborative learning. Here are notes of three groups that reflected on this problem:

- Group A: Our group engaged in a heated discussion about whether to provide students with opportunities to experience and write teaching cases. Each member presented their personal views in an attempt to persuade others. Despite these differences, the SCLP process in our group was generally well-managed. When deviations from the topic or slow progress occurred, the group leader promptly found ways to adapt to the situation.
- Group B: Disagreements arose during our discussions, particularly regarding our understanding of the evaluation criteria. We reached a consensus through negotiation and careful consideration. When progress stalled, we chose to take short breaks to discuss something interesting to refresh our minds and then go back to the collaborative tasks.
- Group C: We encountered a lack of unity in the arrangement of learning activities. However, through negotiation, we reached a unified agreement. Nonetheless, we only conducted a few reflection activities during the SCLP process. Therefore, we had to schedule more additional time for further discussions, which impacted the overall effectiveness of the SCLP to some extent.

Similarly, the disagreements identified by the groups were primarily rooted in the discussions and collaboration among group members. However, the ways of resolving these discrepancies varied. Group A fostered an environment of open and free communication, allowing each member to express individual views and ideas. Group B strategically adapted the intensity of discussions and provided members with time for personal reflection, while Group C arranged additional discussion activities to ensure task completion.

In terms of what needed to be improved for the upcoming round, several participants indicated that they felt inadequately prepared for lesson planning activities in this format. They expressed the need to enhance their skills in language organization and expression, particularly during collaborative discussions and teaching practices. Some participants recognized that they had been more passive throughout the process and expressed a commitment to becoming more proactive and contributing more in future SCLP activities.

### 3.2. The Second Round of SCLP

#### 3.2.1. Changes in the Second Round

Upon completion of the first SCLP procedure, the course instructor assigned the second lesson planning tasks. The designs and research procedures for the second round of SCLP remained largely unchanged, as they closely followed the structure of the first round and were conducted within the same program and setting. One evident change in the second round, however, was the active encouragement from the course instructor and investigator to prompt participants to reconsider the role CSCL tools played and attempt to explore more ways for applying and integrating the tools into the lesson planning and simulated teaching. Another modification involved placing greater emphasis on the initial status and emotional aspects of each participant during task assignments and collaborative efforts.

#### 3.2.2. Shared Task Understanding in the Second Round

At the beginning of the SCLP, the groups re-selected the topics of the lessons. In this round, the time spent by each group discussing was a bit longer than that of the previous round. The discussion process was based on the previous experience of lesson planning and the suggestions of the presentation, in terms of the difficulty, the effectiveness of the completion, and whether the members had the relevant knowledge and interests. Members then began to share and discuss their understanding of the task. In this round, we focused mainly on two groups as case studies to illustrate their collaboration and regulation throughout the SCLP process. The lesson topics chosen by the two groups were *Using Artificial Intelligence Tools to Solve Problems* and *Branch Structure Algorithm*, respectively. A member of Group D mentioned in the *Group Task Understanding Questionnaire*:

- “Each of us proposed a lesson topic individually, and we then discussed and agreed upon the lesson topic for our shared lesson plan.”

Group E additionally considered the technical and teaching environment in relation to teaching practices:

- “We began by exploring the supporting technologies in the teaching environment and deliberating on what would be appropriate to teach given the available tools. The interactive whiteboards in the micro-teaching classrooms offer a space for students to write without contaminating the physical environment, thereby facilitating lesson content delivery.”

Next to the determination of lesson topics, the groups engaged in role and task assignments. They first discussed how to divide the overall lesson planning task into pieces of sub-tasks. Each member demonstrated their strengths and outlined their knowledge or skills. Members of the two groups reported:

- Group D: Member A and Member B in our group excelled in code writing and instructional evaluation. Member C specialized in debugging codes and facilitating interactions in teaching and learning. Member D was skilled in instructional design and presentation, while Member E was proficient in organizing learning activities and tasks. Each member was expected to have foundational subject knowledge as well as knowledge of artificial intelligence.
- Group E: Member A in our group was proficient in searching for and preparing instructional resources. Member B could be a lead instructional designer. Member C specialized in knowledge synthesis and content technology. Member D was good at instructional skills, and Member E was familiar with the application of CSCL tools. We were required to be familiar with the software and hardware in the teaching environment and how to integrate the technologies into teaching. Additionally, we needed to have a deep understanding of the textbook content.

In the previous round of the SCLP, although group members identified their respective interests and self-efficacy, they did not fully consider this information when making decisions. Members randomly chose roles based on personal preferences. In this round, we refined this process by encouraging them to review each member’s expertise for the SCLP prior to task assignment. This approach allowed each participant to choose a role and sub-tasks that best suit their skills and self-efficacy, thereby enhancing the effectiveness of lesson planning and collaborative tasks. At the end of this stage, the group reached an agreement regarding the task characteristics, roles, and responsibilities, as well as the knowledge and skills required for the SCLP.

#### 3.2.3. Shared Plan Making in the Second Round

In this stage, participants developed a more concrete schedule for the entire SCLP procedure. They attempted to monitor their emotional and motivational states in order to implement appropriate regulatory and collaborative learning strategies if passive behaviors arise from other group members. The following discourse illustrates how a leader in Group D brought the members back to the activities when some expressed feelings of boredom and anxiety.

- PT A (Leader): “The only thing we left in the Instructional Resources and Environment section is the hardware device description?”
- PT B: “Right.”
- PT C: “Should we divide the overall task again?”
- PT A: “Yes, let’s make it more detailed.”
- PT B: “I think we set too many learning activities in our plan.”
- PT D: “That is why I think the activities are intensive.”
- PT C: “Yes, the schedule is full. We only have 45 min to teach and these activities may take up much time.”
- PT D: “But I felt very tired now…”
- PT B: “So… what shall we do next?”
- PT A: “Come on everyone! Let us rearrange the tasks and our lesson plans.”
- PT C: “Sure. I believe you two (pointing to PT D and PT E) can take on the redesign work.”

When a member felt that the initial lesson plan was poorly organized and felt a sudden sense of fatigue, the group leader proposed a redesign suggestion and sought to push the SCLP process forward. This is a standard SSRL process. The leader effectively pulled the members’ attention back to the task and group discussion, and moved towards the shared goal.

Overall, during the shared lesson-plan-making stage of this round, there was an improvement in the learning atmosphere, interest and self-efficacy status of the groups compared to the previous round, based on our observations and reports of affective and motivational status, and in the subsequent lesson planning activities. The groups set specific expectations and plans for the delivery of lesson content, collaborative learning strategies, and coordination among members.

#### 3.2.4. Shared Lesson Plan Synergy in the Second Round

Next, participants engaged in discussions and regulatory activities designed to co-construct the shared lesson plans. Each member assumed responsibility for monitoring the participation of others, ensuring the continuity of collaborative learning, and evaluating the outcomes of the group lesson plan to identify potential problems and discrepancies between the current progress and expected goals. Throughout the SCLP procedure, the prospective teachers utilized various types of CSCL tools to create a synergistic process. For example, on the online collaborative documentation platform (Shimo), participants worked together to develop the lesson plans, allowing them to monitor and regulate progress in editing and revising the plans. In one case, Group D had a long discussion on the instructional design of the lesson plan, with one member inquiring whether a related topic should be incorporated into a particular module. The members eventually reached a shared understanding after negotiation:

- PT B: Do you think we should incorporate the two tasks into the Exploration and Practice section (a section in the lesson plan)?
- PT C: I’m not sure. The latter two modules are complex, and it would be more complicated if we add the tasks there.
- PT A: I don’t believe we can articulate the tasks adequately. As future teachers, we lack familiarity with both the teaching content and the underlying mechanisms.
- PT B: Alright…
- PT D: The main issue is that we don’t have enough time to explain these tasks in one lesson.
- PT A: However, the challenge is that we cannot explain these tasks clearly. Experiential tasks are difficult to describe with concrete terms. Students may only gain a superficial understanding. They may fail to fully grasp the tasks after knowing the theoretical explanations. For example, they might understand that an input, such as a photo, can generate an output, but what they really want to know is the process—how do we get from X to Y? Explaining that process thoroughly requires a significant amount of time.
- PT B: Indeed, that is a very difficult task.

During this stage, the groups completed the SCLP task faster through flexible planning and synergy; at the same time, each group also monitored and evaluated the group’s outcomes, collaborative learning process, and members’ feelings and attitudes.

#### 3.2.5. Simulated Teaching and Evaluation in the Second Round

When completing the initial version of the shared lesson plan, each member conducted a simulated teaching practice to elaborate the lesson plan and simultaneously demonstrate their teaching skills and techniques. Unlike the first round, this time, each member was required to create an individual presentation document to aid their individual teaching. Participants were encouraged to refer to the shared lesson plan, teaching practice evaluation criteria, and peer feedback when preparing their presentations. At this stage, the groups systematically evaluated the structure and content of the lesson plans, as well as the behaviors and performances of the group members in the simulated teaching practices.

#### 3.2.6. Outcome Summary and Presentation in the Second Round

Through a cyclical process of planning, practicing, evaluating, and adapting, each group worked out a shared lesson plan that was submitted to the instructor. Then, a representative from each group made a presentation on the basis of the group’s shared lesson plan. When the presenter experienced difficulties in elaborating some key points or sections of the presentation document, and other members provided timely support and additional comments. Participants from other groups observed the presentation and gave immediate feedback to the presenter. The constructive suggestions can significantly help the prospective teachers improve their teaching and facilitate their participation in future SCLP activities.

At the end of this stage, participants reflected on the collaborative learning process and strengthened their awareness of regulatory behaviors and strategies in the lesson planning and simulated teaching. They were asked to write group reflections and summaries of the second round of SCLP. The following part outlines the challenges identified by Groups D and E, as well as the solutions that they implemented:

- Group D: We mainly encountered two challenges. First, we were unfamiliar with some operations of the interactive whiteboard and screen-switching function at the very beginning of SCLP. We attempted to address this challenge via repeated attempts in the micro-teaching classroom and by learning from another group. Second, we experienced disagreements regarding the instructional design (content selection and design intention), the selection of the scope of knowledge planned to be taught, and the key points. We spent a certain amount of time preparing the related knowledge and CSCL tools, and acquired inspiration from other good online cases.
- Group E: We encountered three challenges. First, when we attempted to transfer the PowerPoint slides to the interactive whiteboard, some of the charts displayed with messy codes. Fortunately, the built-in drawing function of the whiteboard helped us solve the problem. Second, in the design stage, we had difficulties selecting the suitable conditions. The continuity and fitness of conditions needed to be carefully considered when designing a branching structure diagram. We made some revisions and organized discussions to accomplish this task. Third, the video designed for the Introduction section was too lengthy. A group member edited the video and picked the piece that perfectly fit with the planned lesson time.

#### 3.2.7. Experiences in the Two Rounds of SCLP

In addition, to explore how prospective teachers perceive the SCLP procedure and its role in supporting teaching practices, five participants were interviewed upon completion of the entire procedure. Each interview consisted of several brief open-ended questions and lasted approximately 5 to 10 min. The first question was about the potential impact of the SCLP on teaching skills and lesson planning. Two participants responded as follows:

- “I definitely believe the SCLP is beneficial to our teaching and lesson planning, as it allows us to actively engage with other members and receive immediate feedback to improve our teaching skills and techniques.”
- “Overall, the SCLP activities enhanced my teaching abilities and the application of CSCL tools in teaching. Initially, I had a limited understanding of the teaching content, which affected my participation in the lesson planning activities. However, as I became more familiar with the content and relevant materials, I felt more confident in contributing to the lesson planning and simulated teaching.”

In addition to recognizing the positive impact of the SCLP on the integration of teaching skills and technology, the second participant described their progress in adapting to the SCLP activities. Not all prospective teachers were able to get used to this new lesson planning approach in a limited time, highlighting the necessity and importance of regulation of learning in this experience.

The next question focused on the collaboration atmosphere and its impact on the SCLP. The interviewers responded positively:

- “The atmosphere for collaboration was relaxed and harmonious, which encourages me to express my viewpoints more freely. A comfortable collaborative learning environment can definitely promote the interaction and communication among members and can improve the effectiveness of the SCLP.”
- “The atmosphere was both lively and orderly. It motivates us to generate new ideas and opinions, and facilitated our progress for completing the tasks and lesson planning.”

The third question was about the participants’ attitudes towards the application of CSCL tools in teaching practices and lesson planning activities:

- “We actively applied some CSCL tools in the SCLP. However, we also thoughtfully considered the impact of the tools on our teaching and the need to use them. While the effective use of the tools can improve our efficiency for lesson planning and the teaching and learning, this is contingent upon mastering the technical aspects of the tools. Without such proficiency, the application and integration of CSCL tools could have a negative effect on both teaching and learning performance.”
- “Personally, I advocate the integration of instructional technology. As a future information technology teacher, it is important to utilize CSCL tools appropriately in teaching and learning activities and to continuously seek more effective ways to apply and integrate them successfully into teaching and learning practices.”

## 4. Discussion and Conclusions

The current study explored the effectiveness of the Shared Collaborative Lesson Planning (SCLP) procedure in enhancing prospective teachers’ professional development through the aspect of Socially Shared Regulation of Learning (SSRL). The findings from two rounds of SCLP activities involving groups of prospective teachers revealed significant insights into the collaborative learning process, regulatory behaviors, and the role of CSCL tools in facilitating effective lesson planning. Overall, the SCLP procedure demonstrated its potential in fostering a collaborative learning environment that supports the development of teaching skills and lesson planning competencies. The cyclical structure of SCLP, shared task understanding, lesson plan making, synergy, simulated teaching, and outcome presentation provided a structured yet flexible framework for prospective teachers to engage in regulation of learning. This process not only enhanced their understanding of teaching content, but also promoted critical reflection and adaptation through continuous feedback and discussions.

The SCLP procedure can play a role in fostering a collaborative environment, particularly in lesson planning activities. The prospective teachers experienced a relatively high level of effectiveness and efficiency during these activities. This suggests that intentional, well-structured regulation of learning is necessary to promote collaboration among teachers, which is consistent with the conclusions reached by Alloway (2014) and Huang et al. (2024). The structured approach offered by SCLP has proven to be an effective way to overcome challenges typically associated with collaborative planning efforts in teacher education.

This study provides a detailed case for incorporating the regulation of learning approaches into teacher education courses. The participants’ interviews indicate that such a procedure provides prospective teachers with valuable opportunities for professional growth and skill development. It has been argued that teachers’ professional development can be anchored through becoming the learners and facilitators of regulation of learning (Dignath & Veenman, 2021; Ortube et al., 2024). Tondeur et al. (2020) also stated that teacher education programs should include collaborative learning to ensure that prospective teachers are well-prepared for their future roles in the classroom. By focusing on collaboration, these programs can better equip teachers with the tools they need to create more effective and engaging learning environments.

The study also delves into a prominent challenge within the field of collaborative learning: ensuring that appropriate support systems are in place to enable productive collaboration among prospective teachers. As Nadiyah and Faaizah (2015) noted, without adequate organizational support, collaborative learning efforts can often fall short. The SCLP procedure has the potential to narrow this gap by creating a learning and teaching environment that is both efficient and conducive to collaboration. In addition, it attempts to solve problems such as the limited time for collaboration and the possibility of participant disengagement, as highlighted in the studies of Rakıcıoğlu-Söylemez et al. (2018) and Tondeur et al. (2020). The structured approach provided by the procedure allows prospective teachers to engage meaningfully with each other and helps them focus on productive lesson planning.

Additionally, the integration and application of CSCL tools can benefit teaching and learning (König et al., 2024), and has further improved the effectiveness of collaborative lesson planning. By using such tools, the prospective teachers are provided with opportunities not only to collaborate with peers, but also to engage in shared decision-making and receive instant feedback. This technological component fosters a more dynamic and responsive learning environment (Hernández-Sellés et al., 2019; L. Zheng et al., 2023). Notably, the use of CSCL tools must take into account specific contexts and the level of applicability. For instance, in the second SCLP round, although we reminded the participants of utilizing the CSCL tools in the collaborative lesson planning processes, they expressed in the interview the need to ”thoughtfully consider the impact of the tools on our teaching and the need to use them.”

Another important aspect of this study is the emphasis on SSRL. This concept has been reinforced in previous research, which implies the crucial role of collective regulatory processes and strategies in collaborative learning (Järvelä et al., 2013; Ucan & Webb, 2015; Y. Zheng et al., 2024). According to Hadwin et al. (2017), SSRL not only supports individual learning, but also strengthens group cohesion and collaboration. The study suggests that the prospective teachers benefit significantly from scaffolded collaborative activities, which do more than just improve their teaching skills, but also contribute to a positive learning culture (Kukkonen et al., 2016). Such activities foster a collaborative mindset that extends beyond lesson planning and encourages their professional development.

Although the present study enhances the understanding of the prospective teachers’ collaborative learning and regulatory behaviors in lesson planning activities, it is subject to several limitations. The first limitation concerns the specific context in which the study was conducted. The research took place in micro-teaching classrooms at a prestigious normal university that was equipped with advanced modern educational technology tools. As a result, the prospective teachers had easy access to emerging technologies and had the opportunity to utilize these technologies in the lesson planning and teaching practices. However, not all teacher education programs can be equipped with such technology-enriched environments. The second limitation pertains to the relatively small sample size, with only 24 prospective teachers from five groups enrolled in the study. All participants were from the same graduate program and had established relationships with their peers, which means that their regulatory behaviors and strategies may have differed significantly from those of teachers with diverse educational backgrounds, teaching experiences, and levels of technological proficiencies. Consequently, this limitation may affect the generalizability of the findings. The third limitation involves the group size, with four or five students per group. Research has shown that the nature of collaborative learning is influenced by group size, and larger groups may experience challenges such as free-riding, interaction difficulties, and coordination issues (Noroozi et al., 2013; Zhang et al., 2021).

Future research can focus on exploring the long-term effects of the SCLP procedure on prospective teachers’ professional development and teaching practices. Longitudinal studies will offer valuable insights into the ongoing benefits of collaborative lesson planning, particularly as it relates to long-term teacher growth. Additionally, it would be valuable to investigate how the SCLP procedure performs in different educational contexts, including different subject areas and grade levels. Such studies could provide a better understanding of how adaptable and transferable the procedure is across diverse teaching and learning environments. Teacher education programs can also benefit from these insights by refining their approaches to collaborative lesson planning and ensuring that future teachers gain the teaching skills and regulatory strategies necessary for success in their careers.

## Figures and Tables

**Table 1:** A participant’s response to group interaction monitoring template.

Questions	Member 1	Member 2	Member 3	Member 4
Cognitive Dimension				
During the shared collaborative lesson planning activities, who did you share your knowledge and skills with?	*	*	*	*
Who evaluated your work or provided suggestions to you when finishing the task?	*	*	*	*
Who did you talk to when you have difficulty during the activities?	*	*	*	*
Whose suggestions are useful during the activities?		*	*	*
Metacognitive Dimension (Stage)				
During the shared collaborative lesson planning activities, who did you discuss with on the task requirements? (task understanding)	*	*	*	*
Who did you work with to construct the task schedule? (planning)	*	*	*	*
Who did you discuss with to set up the task assignments? (task assignment)	*	*	*	*
Who did you discuss or share with about information of the progress of group works? (monitoring)	*	*	*	*
Who did you discuss with to resolve problems emerged in the process of shared collaborative lesson planning? (adaptation)	*	*	*	*
After the shared collaborative lesson planning activities, who did you work with to do the reflection? (reflection)		*	*	
Motivational/Emotional Dimension				
During the shared collaborative lesson planning, whose ideas or works did you appreciate?		*	*	
Who ignored your ideas or suggestions?				
Who opposed or reluctant to support your ideas or works?	*			

Note: * means the participant believed that the member exhibited the described behavior.

## Data Availability

Data are available upon request.

## Appendix A. Group Task Understanding Questionnaire

1. What lesson topics do you chose for collaborative lesson planning?
2. How do you reach a consensus when choosing the group lesson topic?
3. What strengths do your group members demonstrate?
4. Please indicate the specific tasks assigned to each member.
5. What knowledge and skills are needed for the lesson topic and content of the shared lesson plan?
6. What available CSCL tools and teaching resources do you plan to apply to complete the tasks?
7. What knowledge and skills do you believe are necessary to successfully complete the shared lesson plan?
8. What challenges do you anticipate encountering with your group members during the shared collaborative lesson planning?

## Appendix B. Individual Initial Status Questionnaire

1. Are you interested in participating in the shared collaborative lesson planning activities?
2. Please explain the reasons that you are interested or not interested in the activities.
3. Please estimate of the possibility of your successful completion of all shared collaborative lesson planning activities.
  A. 0–20%	B. 20–40%	C. 40–60%	D. 60–80%	E. 80–100%
4. What type of collaborative environment do you expect to be conducive to effective collaborative lesson planning?
5. What basic knowledge and skills are considered necessary for effective shared collaborative lesson planning?
6. Do you believe you already possess the knowledge and skills? What aspects do you think need improvement?

## Appendix C. Reflection on the SCLP Procedure

1. What challenges did you face during the shared collaborative lesson planning, and how did you address them?
2. Did you modify the task understanding and goal setting during the initial stage of the shared collaborative lesson planning? How did you make adaptations?
3. Were there any disagreements when collaborating with group members? How did you handle these conflicts?
4. Did you reflect on your performance in the shared collaborative lesson planning (e.g., your participation, task implementation, communication with other members)? Did you make any adaptations?
5. Was the participation in task discussion and collaboration evenly distributed among all group members?
6. Do you believe it is necessary to apply and integrate CSCL tools into the lesson plans and simulated teaching? Do these tools facilitate or hinder the shared collaborative lesson planning activities?
7. Please provide a comprehensive evaluation of your performance. What areas do you believe need improvement for future collaborative lesson planning activities?
